# Hierarchical neural processing in γ oscillations for syntactic and semantic operations accounts for first- and second-language epistemology

**DOI:** 10.3389/fnhum.2024.1372909

**Published:** 2024-09-23

**Authors:** Laurent Dekydtspotter, A. Kate Miller, Kyle Swanson, Jih-Ho Cha, Yanyu Xiong, Jae-Hyun Ahn, Jane A. Gilbert, Decker Pope, Mike Iverson, Kent Meinert

**Affiliations:** ^1^Department of French & Italian, Indiana University, Bloomington, IN, United States; ^2^Department of Second Language Studies, Indiana University, Bloomington, IN, United States; ^3^Department of World Languages and Cultures, Indiana University–Indianapolis, Indianapolis, IN, United States; ^4^Oral English Proficiency Program, Purdue University, West Lafayette, IN, United States; ^5^Alabama Life Research Institute, University of Alabama, Tuscaloosa, AL, United States; ^6^Cognitive Science Program, Indiana University, Bloomington, IN, United States

**Keywords:** processing, movement, anaphora, time-frequency, EEG

## Abstract

**Introduction:**

We discuss event-related power differences (ERPDs) in low- and broadband-γ oscillations as the embedded-clause edge is processed in *wh*-dependencies such as *Which decision regarding/about him/her did Paul **say that** Lydie rejected without hesitation?* in first (L1) and second language (L2) French speakers.

**Methods:**

The experimental conditions manipulated whether pronouns appeared in modifiers (Mods; *regarding him/her*) or in noun complements (Comps; *about him/her*) and whether they matched or mismatched a matrix-clause subject in gender.

**Results:**

Across L1 and L2 speakers, we found that anaphora-linked ERPDs for Mods vs. Comps in evoked power first arose in low γ and then in broadband γ. Referential elements first seem to be retrieved from working memory by narrowband processes in low γ and then referential identification seems to be computed in broadband-γ output. Interactions between discourse- and syntax-based referential processes for the Mods vs. Comps in these ERPDs furthermore suggest that multidomain γ-band processing enables a range of elementary operations for discourse and semantic interpretation.

**Discussion:**

We argue that a multidomain mechanism enabling operations conditioned by the syntactic and semantic nature of the elements processed interacts with local brain microcircuits representing features and feature sets that have been established in L1 or L2 acquisition, accounting for a single language epistemology across learning contexts.

## Introduction

Language relies on an extensive cortical network involving both specialized and multidomain cognitive control regions (e.g., Fedorenko and Thompson-Schill, 2014; Hagoort, 2014; Hertrich et al., 2020) in both first- (L1) and second-language (L2) speakers (Mitsuhashi et al., 2020; Yokoyama et al., 2006). In multilingual brains, L1 and L2 constitute overlapping neurofunctional subsystems within the language network (Paradis, 2009). As Green and Abutalebi (2007) pointed out, “on the simplest assumption the acquisition of a second language will utilize existing devices. The processing of its lexical, grammatical/morphological properties and its prosody will lead to its representation in a network shared with L1 (Green, 2003, 2005; Green et al., 2006)” (p. 560). The preconfigured subnetwork for the L1 accounts for the initial state of L2 acquisition. Adaptive neuronal ensembles for new features and local circuits for feature bundles within this neurofunctional space for language account for language development.

Hierarchical processes in the creation of syntax-semantics (syn-sem) objects that are then turned into sequential phrasal arrays for production account for implicit (interpretive) knowledge of the L2 beyond the L1 even in the absence of explicit instruction (Dekydtspotter, 2001; Lardiere, 2009; Schwartz and Sprouse, 1996; *inter alia*). Hence, similarities between L1 and L2 would have their neurocognitive bases in the processes instantiating the construction of basic language objects and their integration into structures. In contrast, differences between L1 and L2 would have their neurocognitive roots in the local neuronal ensembles for features and circuits for feature bundles established in initial L1 or later L2 development, affecting downstream processes. Others also suggest delayed computations of lexico-grammatical objects and relations in shallow structuring (Clahsen and Felser, 2018), non-predictive processing (Covey et al., 2024), and interference from working memory (Cunnings, 2017a,b) as major differences between L1 and L2 processing.

Differences have been found in event-related potentials (ERPs) in response to (L1-based) number vs. (new) gender agreement in English speakers’ acquisition of Spanish (Gabriele et al., 2013), echoing behavioral effects in timed tasks (Lopéz Prego and Gabriele, 2014). Thus, in the L2 acquisition of Spanish by L1-English learners, Gabriele et al. (2013) reported that adjective–noun gender agreement violations resulted in a P600 effect only at high proficiency levels. In contrast, adjective–noun number agreement violations resulted in P600 effects at both low and high proficiency levels. Similarly, for German-speaking intermediate learners of Dutch, Lemhöfer et al. (2014) reported a P600 response to gender agreement violations on determiners, but only when participants’ own (sometimes non-target-like) gender representations were considered.

Here, we argue that the study of oscillations provides complementary information of greater granularity (Poeppel and Embick, 2017) that can address different neurocognitive subprocesses in L1 and L2. Hence, the maintenance of sentential information enabling prediction implicates synchronizations of cell assemblies in the beta (β, 13–30 Hz) rhythm. Investigating β power in such synchronizations, Dekydtspotter et al. (2023) addressed the questions of timing and resources in L1 vs. L2 for anaphoric relations linked to modifier (Mod) vs. complement (Comp) structures of *wh*-fillers in bi-clausal cyclic *wh*-dependencies in French. Anaphora-linked event-related power differences (ERPDs) modulated by *wh*-filler Mod and Comp structures were found in advance of access to the bridge verb retrieval in predictive processing at the intermediate gap site and at the subordinator for an embedded-clause *wh-*dependency in L1 and L2. Additional cell assemblies were implicated in these computations in L2, but similar timing was observed across L1 and L2. β oscillations can thus address aspects of top-down predictive processing and resource allocations in ongoing maintenance of L2 processing but cannot address how basic operations are implemented.

We show that addressing the implementation of basic operations in L1 and L2 requires examining oscillatory activity in the higher gamma (γ, > 30 Hz) band as cell ensembles form in cortical output. Kazanina and Tavano (2023c) and Murphy (2024) present neurocognitive hypotheses about the nature of syntax in the brain involving a processing loop implicating both structures in slow rhythms and object construction in fast rhythms. Therefore, building on Dekydtspotter et al. (2023), we examined γ-band activity in evoked power as cell assemblies form to create anaphoric chain objects in fast rhythms during the processing of cyclic *wh*-dependencies in L1 and L2 as a *wh*-filler is silently integrated into an embedded clause (Chomsky, 1977). We report anaphora-linked ERPDs at the embedded-clause edge for Mods such as *regarding him/her* vs. Comps such as *about him/her* despite no differences in sequential arrays at the clause edge in dependencies like *Which decision regarding/about him/her did Paul say that Lydia rejected?* The paper is organized as follows: We first provide an overview of oscillations in language. We then discuss the role of γ oscillations in the neural code for language, before presenting the current study, our research questions and hypotheses, and testing and analysis procedures. Following the presentation of our results, the discussion returns to the role of γ in basic syntactic and semantic operations and how the study of oscillations provides additional insight into the nature of language in the brain, whether acquired as an L1 or implicitly learned as an L2.

### Oscillations in language

A body of work on neural oscillations—that is, the “discrete and collective activity of neurons (spiking, bursting, post-synaptic potentials etc.) that … operate as a cohesive functional network” (Giraud, 2020, p. 1106)—seeks to characterize language processes in terms of synchronized oscillations between cell assemblies in distinct rhythms. In adults, these frequencies—namely, delta (δ, 0.5–4 Hz), theta (θ, 4–8 Hz), alpha (α, 8–13 Hz), beta (β, 13–30 Hz), and gamma (γ, >30 Hz)—are found to be engaged in support of different processes in speech and language (Arnal and Giraud, 2012; Bastiaansen and Hagoort, 2015; Ding et al., 2016; Giraud and Poeppel, 2012; Hasson et al., 2015; Kazanina and Tavano, 2023b; Lewis et al., 2015, 2016b, 2023; Meyer, 2018; Murphy, 2015, 2021, 2024; Prystauka and Lewis, 2019; Weiss and Mueller, 2012; *inter alia*). However, the neural mechanisms and the specific functions of these distinct neural oscillatory rhythms in structure building are still highly debated (Coopmans et al., 2023; Ding, 2022; Kazanina and Tavano, 2023b,c; Meyer et al., 2019; Lo et al., 2023). It has been claimed that α generally mediates the (dis)engagement of cell assemblies to manage the focus of resources (Payne and Sekuler, 2014) and that β manages the maintenance of the sentence-level cognitive set, enabling prediction (Lewis et al., 2023). γ has been linked to the “match between top–down prediction and bottom–up input” (Wang et al., 2018, p. 1083). It has also been linked to the encoding and retrieval of items (Gruber et al., 2004) and, crucially, both combinatorial (Artoni et al., 2019; Nelson et al., 2017) and interpretive (Fedorenko et al., 2016; Nieuwland and Martin, 2017; Peña and Melloni, 2012) operations. In syntactic processing, the computation of phrases and structural configurations has been linked to δ oscillations (Ding, 2022; Meyer et al., 2019). The computation of syntactic elements into basic hierarchical objects has also been linked to γ activity (Artoni et al., 2019; Nelson et al., 2017).

Taking a bottom-up phrasal-array approach to language, Meyer et al. (2019) argued that δ oscillations implement syntactic chunking and entrain other rhythms. In this view, θ supports memory retrieval, α the storing of phrases, β prediction, and γ goodness of fit as well as semantic integration. Clearly, much remains to be fully understood. In view of γ effects reflecting syntactic combination (Nelson et al., 2017), Kazanina and Tavano (2023c) postulated a central role for γ oscillations in the computation of basic hierarchical syn-sem objects, calling for investigations of the γ range. Indeed, most of the empirical research thus far has addressed the role of γ oscillations in reaction to distinct phrasal arrays in the input in terms of syntactic or semantic integration. However, new proposals for the role of γ oscillations in circuitry implementing basic operations (Kazanina and Tavano, 2023c; Murphy, 2024) predict that such γ oscillations should be detectable in power differences when top-down computations satisfying lexico-grammatical needs, such as the need for the identification of variables in syntax or discourse, are at play. The role of γ oscillations in the implementation of basic operations seems therefore best established in γ power differences obtained without differences in sequential arrays in the input concurrent with these processes so that the input-based entrainment of fast rhythms can be excluded as a possible account for these differences.

Addressing the epistemological status of L1 and L2 in terms of aspects of the construction of basic language objects for representations involving local neuronal ensembles and circuits established in initial L1 or later L2 development requires documenting γ oscillations in the creation of new objects. Taking a top-down approach to syn-sem object creation, Murphy (2024) postulated a neurofunctional Representation Operation Structure Encoding (ROSE) loop architecture for syntax in which cortical circuitry for operations enables the formation of basic hierarchical syn-sem objects in broadband γ oscillatory activity. Thus, cortical circuitry in the temporal cortex implements a hierarchical γ operational workspace: “the component of ROSE responsible for manipulating and combining R [representational] units rather than simply accessing them” (Murphy, 2024, p. 7). In spike-local field potential phase coupling, syn-sem features are composed into manipulable feature sets enabling elemental units to enter the operational workspace in high γ. Workspace computations for syn-sem objects involve neuronal assemblies for lower and larger representational levels in broadband-γ oscillatory processing. γ-Constructed syn-sem objects are then integrated into structures and maintained in item memory in multiplexed interactions between cortical sites synchronized in low γ and subcortical sites that are synchronized in θ for item memory and δ for structural memory.

### Neural signals and anaphoric computations

Murphy’s (2024) ROSE proposal echoes a referential cortical-hippocampal processing loop in structure-based and discourse-based anaphora processing put forth by Nieuwland and Martin (2017) and Coopmans and Nieuwland (2020). In processing English sentences such as *The boy_i_ thought that he_i_/she would win the race*, the pronoun *he* can immediately be anaphorically interpreted as *the boy*, as represented by co-indexing. A deictic interpretation of the pronoun *he* with *the boy* is also still available, although the anaphoric interpretation of *he* seems favored as another referent does not need to be accommodated. In contrast, the pronoun *she* does not have a sentential antecedent and must be eventually interpreted deictically by accommodating a contextual referent. Nieuwland and Martin (2017, Experiment 4) reported that the anaphoric condition in which *he* could be bound to *the boy* resulted in greater power than the deictic condition, first in low γ (40 Hz) about 500 ms after the pronoun was encountered, followed by effects at approximately 60–80 Hz. Nieuwland and Martin argued that these power asymmetries in γ signaled the anaphoric integration of the matching pronoun in the ongoing sentence interpretation in semantic unification, in contrast to the non-matching pronoun *she*. Similar ERPDs were also obtained in Dutch sentences akin to *Jim_i_ told Mary/James that he_i_ was a bit promiscuous* (Nieuwland and Martin, 2017, Experiment 1). When the pronoun *he* could be unambiguously bound to *Jim,* greater γ power was induced than in the anaphorically ambiguous condition. Nieuwland and Martin characterized these effects in γ power as implementing the resolution of the anaphoric interpretation of the pronoun, which seems consistent with a bottom-up approach where γ power signals best fit in semantic integration. However, the linguistics of pronouns as variables in need of contextual values, unlike constants with lexical values, also requires top-down interpretive processes: Variables must receive referential values that are either syntactically determined through binding or else assigned in discourse, with a determination in syntax avoiding a referential value assignment in discourse (Reuland, 2001). Therefore, the fact that γ oscillations arise in timing with distinct arrays in the input still does not exclude a top-down process in which γ oscillations implement basic referential processes responding to general syn-sem principles (Koornneef and Reuland, 2016; Reuland, 2001).

**Figure 1:**
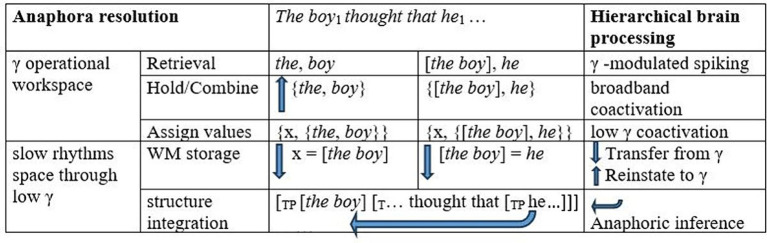
Neurofunctional processes in structure-based anaphora resolution. WM = working memory. Arrows going up indicate the coactivation of cell ensembles for representations in gamma as is required in reinstatement. Arrows going down indicate interactions with slow rhythms.

Nieuwland and Martin argued that the low-γ (40 Hz)/high-γ (60–80 Hz) patterns arose as low-γ activity reinstated referential elements into a γ operational workspace for unification/integration in high γ. Under a linguistic understanding of the nature of anaphoric expressions as variables inherently dependent on syntactic or discourse contexts, these effects in γ power can be tied to the formation of a referential chain for *the boy* and *he* in binding (see Chomsky, 1981, among many others, as well as discussion below). In binding, bound expressions receive a single referential value in one fell swoop, that is, as a single syntactic object (Koornneef and Reuland, 2016). Binding involves a referential chain syntactic object that implements referential identification. Hence, the broadband-γ effects found in anaphora resolution must, *inter alia*, signal such referential processes. We therefore consider the computation of such a syntactic chain object in a hierarchical γ operational workspace that implements basic operations, including anaphora resolution through binding, upon the transfer of referential elements for referential computations in low γ. Within the ROSE model framework, the referential computations as *he* is encountered in *The boy_i_ thought that he_i_ would win the race* would be as in Figure 1.

As modeled in Figure 1, the referential interpretation of *he* as *the boy* presumably requires several cycles of hierarchical computations for basic objects in γ, first for the combination *the boy* and then for the combination *the boy*…*he*. As illustrated in the third column, when the expression *boy* would first be accessed, *the* and *boy* would be combined in γ as argued in Murphy (2024). The object {*the*, *boy*} would be formed, and this combination would be assigned a discourse referent, forming the interpretive object {x, {*the*, *boy*}}. The interpretive object identifying *the boy* with a discourse referent (x = [*the boy*]) and the labeled syntactic object for the expression *the boy* {the, {the, boy}} both require transfer to item and structural memory to allow for a (partial) interpreted sentential structure to arise for the unfolding sentence. Within a cortical γ operational workspace implementing basic syn-sem operations, therefore, as soon *he* is structurally integrated into the ongoing structure, the units for *the boy* and *he* must be reinstated from working memory to create a referential chain {x, {*[the boy], he*}}, with the discourse referent for *the boy* as its referential value. The proposed steps implementing syntactic binding in γ offer a finer-grained, linguistically motivated hypothesis for the exploration of the role of γ oscillations in complex syntactic dependencies. Crucially, we argue that, in the phenomenon of reconstruction in filler-gap dependencies, in which sentence-initial filler expressions are interpreted in terms of later-derived gap positions, basic referential chain formation processes reflecting top-down general principles of language interpretation can be tied to implementation in γ without the possibility of entrainment by concurrent sequential arrays.

### The study: comps vs. mods in anaphora resolution

Using the paradigm in (1a-d), Dekydtspotter et al. (2023) examined induced oscillatory responses that were time-locked but non-phase-locked to stimuli from 5 to 60 Hz at the bridge verb and subordinator, reporting results in the β rhythm linked to prediction. Given a role for γ oscillations in the implementation of binding, broadband-γ effects in evoked activity as cell assemblies form across L1 and L2 status could address delayed computations, and narrowband-γ effects across L1 and L2 status could speak to retrieval. We therefore turn to evidence of syntax-dependent anaphoric relations computed in the γ syn-sem operational workspace.

Critical items like (1a-d) manipulated the contents of the *wh*-filler, with either a Mod (1a, c) or a Comp (1b, d) qualification of the noun, and they also varied whether the pronoun inside the *wh*-filler found a gender-matching antecedent in the matrix clause (1a, b) or embedded clause (1c, d).

(1a)*Quelle décision le concernant est-ce que Paul a dit que*

which decision him regarding is-it that Paul has said that

*Lydie avait rejetée sans hésitation?*

Lydie had rejected without hesitation

‘Which decision regarding him did Paul say that Lydie had rejected without hesitation?’

(1b)*Quelle décision à propos de lui est-ce que Paul a dit que*

which decision at words of him is-it that Paul has said that

*Lydie avait rejetée sans hésitation?*

Lydie had rejected without hesitation

‘Which decision about him did Paul say that Lydie had rejected without hesitation?’

(1c)*Quelle décision le concernant est-ce que Lydie a dit que*

which decision him regarding is-it that Lydie has said that

*Paul avait rejetée sans hésitation?*

Paul had rejected without hesitation

‘Which decision regarding him did Lydie say that Paul had rejected without hesitation?’

(1d)*Quelle décision à propos de lui est-ce que Lydie a dit que*

which decision at words of him is-it that Lydie has said that

*Paul avait rejetée sans hésitation?*

Paul had rejected without hesitation

‘Which decision about him did Lydie say that Paul had rejected without hesitation?’

*Wh*-fillers are argued to be re-represented as unpronounced copies at intermediate and tail positions in such dependencies, with gaps signaled here as *wh*-filler traces (*t*) in the bracketed representations in (2a, b). Crucially, Mods and Comps are syntactically, and therefore also interpretively, distinct. A range of accounts have been proposed (see Chomsky, 1995, 2020; Lebeaux, 1988, 2000; and Milway, 2022, for recent discussions).

(2a)[[quelle <décision, le concernant>] [est-ce que [Paul a dit [*t* [que Lydie avait rejetée *t* sans hésitation]]]]].

(2b)[[quelle {décision, à propos de lui}] [est-ce que [Paul a dit [*t* [que Lydie avait rejetée *t* sans hésitation]]]]].

For Chomsky (2020), Comps involve set-Merge (as in {*decision*, *about him*}, with the noun assigned as head {*decision*, {*decision*, *about him*}}), linked to saturation and semantic function-argument relations. A Mod instead involves a linearly ordered pair-Merge into a sequence [<*decision*, *regarding him* > definable as {{*decision*}, {*decision*, *regarding him*}}, following Kuratowski (1921)], with a conjunctive interpretation. The adjunct is, therefore, “off in a different dimension” (Chomsky, 2020, p. 50). Hence, in addition to circuitry in the temporal cortex implementing a hierarchical operation workspace for set-Merge (Murphy, 2024), the pair-Merge process for Mods requires an ordering workspace with distinct circuitry. Such a workspace is independently needed for the sequenced spell-out of structural hierarchies in language production (Matchin and Hickok, 2020). These distinct processes for Mods and Comps are encoded in the bracketed representations in (2a, b).

For Lebeaux (2000) and Milway (2022) both, Comps involve Merge and, specifically, the projection of lexical elements into the phrasal structure. For Lebeaux, however, Mods involve an Adjoin operation, which applies to tree structures, blending them together to enable linearization. Milway offered an alternative account where Mods involve two parallel derivations that unify only in the process of externalization. Crucially, across these characterizations, the adjunct is “off in a different dimension” (Chomsky, 2020, p. 50), which entails a different process of anaphora resolution for pronouns inside them.

Hence, at one level, the preposition *à* in (1b, d) is lexically required by the noun *décision* and cues a lexically selected complement. The noun is thus construed as relational: It maps an entity to a decision related to this entity. The Comp is lexically required by the head noun at each processing stage. With a Mod such as *le concernant* “regarding him” (1a, c), the non-relational noun signals that a decision was made, with the conjunct providing additional information. Moving to the next level, the anaphoric interpretation of pronouns is subject to an anti-locality syntactic condition: Pronouns must be free in a local domain containing a subject as per Binding Condition B for them to be interpreted as referring to a syntactic antecedent (Chomsky, 1981).

With Mods (1a, c), the clausal modifier constitutes a local domain for the anaphoric resolution of the pronoun. Therefore, the pronoun is immediately associated with an as-yet-unidentified discourse referent, and it can later be identified with a matching antecedent via discourse coreference. With lexically selected Comps (1b, d), the *wh*-dependency instead enables pronominal binding in which the pronoun is syntactically and semantically dependent on a gender-matched c-commanding antecedent. As Chomsky (1981) pointed out, sentences such as *John repeated a story about himself, w*ith the anaphor *himself* bound by *John*, show the clause to constitute the default binding domain. To note, a nominal can constitute a binding domain only if it has a nominal subject, such as a possessor. Hence, in *John repeated Bill’s story about himself*, the anaphor *himself* depends on *Bill* for its interpretation. In contrast, a pronoun like *him* in *John repeated Bill’s story about him* is valued outside the nominal. *John repeated a story about him* is also possible with *John* as a binder for the pronoun *him*. However, in this case, the story must originate with someone other than John. Chomsky argued that in such cases, a silent nominal subject, identified as PRO, allows a nominal binding domain of last resort, i.e., *John_i_ repeated a PRO*_j_ *story about him*_i_. Examining processing costs linked to anaphors vs. pronouns in bi-clausal filler-gap dependencies in L1 and L2 speakers, Dekydtspotter et al. (2012) provided real-time behavioral evidence that clauses provide the default binding domain and that a nominal subject for a *wh*-filler is computed only when the default option is unavailable.

Empirical evidence consistent with the computation of *wh*-filler representations in intermediate gap positions among L1 speakers has been found in experiments using priming, pupillometry, and self-paced reading (Fernandez et al., 2018; Gibson and Warren, 2004; Keine, 2020; Miller, 2015; Pliatsikas and Marinis, 2013). When it comes to L2 speakers, there is also evidence from at least a subset of studies (Dekydspotter and Miller, 2013; Fernandez et al., 2018; Miller, 2015; Pliatsikas and Marinis, 2013; Pliatsikas et al., 2017) for clause-edge *wh*-filler re-representation.

As Coopmans and Nieuwland (2020) and Nieuwland and Martin (2017) demonstrated, γ oscillations can be linked to the retrieval of referential elements and semantic unification, which includes syntactic binding in anaphora resolution (Chomsky, 1981). The subprocesses necessary to account for the standard linguistic model of binding could be implemented by a hierarchical syn-sem operational workspace as hypothesized in Figure 1. Hence, subprocesses forming chain objects and assigning references would characterize the nature of broadband effects. In such a workspace that interacts with slow rhythms, γ oscillations are engaged in hierarchical processes beyond semantic unification for sequenced lexico-syntactic phrase-structure arrays that have been chunked in δ (Ding, 2022; Meyer et al., 2019). In top-down predictive processing, unpronounced copies of *wh*-fillers including pronouns are (silently) re-represented structurally, necessitating that referential chain objects for binding also be computed between antecedents of various sizes and pronouns in covert syntax, as they must in overt syntax. These syn-sem computations presumably take place in γ, given the results in Nieuwland and Martin (2017).

**Figure 2:**
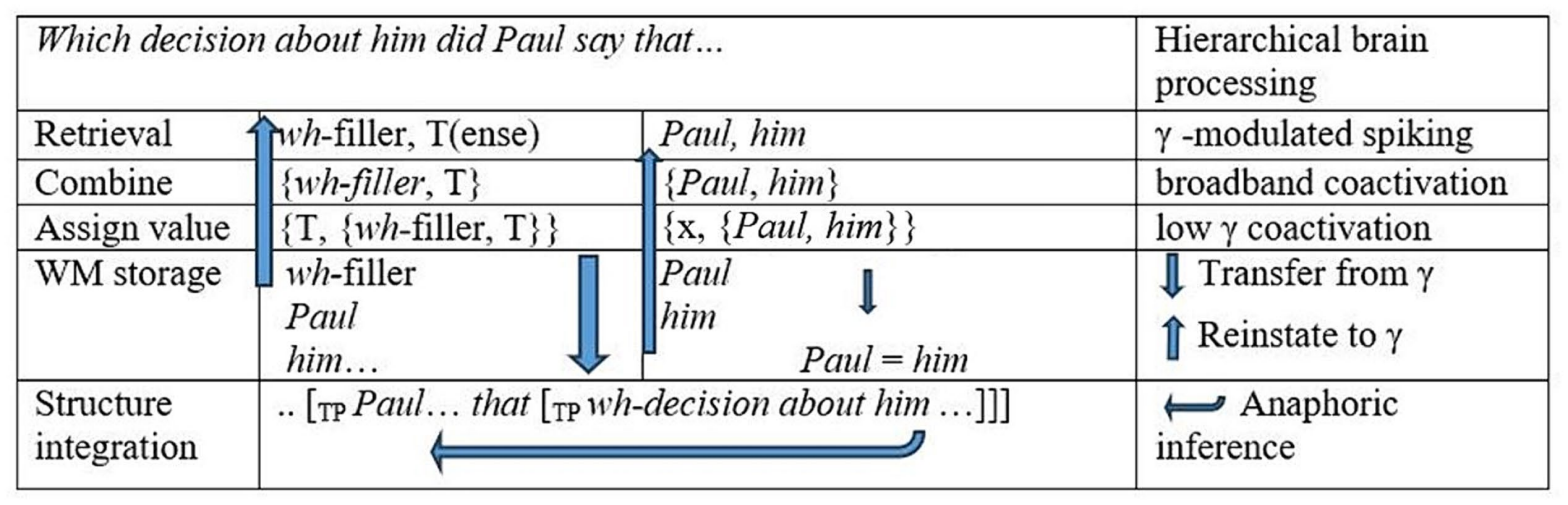
Dynamic processes in structure-based anaphora resolution into the embedded clause. WM = working memory. Arrows going up indicate the coactivation of cell ensembles for representations in gamma as is required in reinstatement. Arrows going down indicate interactions with slow rhythms.

In our manipulation, whether or not the matrix-clause antecedent matches the pronoun inside the *wh*-filler should further affect γ-range ERPDs in a way that highlights the role of syntactic binding. In top-down processing of *wh*-filler-gap sentences like *Which decision about him did Paul say that Lydia rejected?*, the subordinator *that* introduces a tensed embedded clause, and we hypothesize that attendant processes of retrieval, chain formation, and referential value assignment will be signaled in narrowband- and broadband-γ effects. These processes are modeled in Figure 2. Coactivations of cell assemblies yielding broadband-γ effects could arise as referential elements *Paul* and *him* are combined as in {*Paul*, *him*} and as this combination is assigned a discourse referent x for an entity named Paul (third column, Figure 2). Hence, as a *wh*-filler copy is re-merged with a clausal tense category {T, {*wh*-filler, T}} and as this object is integrated into a structure (second column, Figure 2) that defines a binding domain and c-command relation between *Paul* and *him* (bottom row, Figure 2), a γ syn-sem workspace implements syntactic binding.

In contrast, in *Which decision regarding him did Paul say that Lydia rejected?*, the pronoun *him* is in a clausal Mod structure, so the Mod structure constitutes a clause defining a binding domain. Hence, a variable discourse referent x can be assigned to the pronoun *him* at the start of the sentence. When the expression *Paul* is encountered in the subject position, this discourse referent for *him* can be identified with the discourse referent for Paul, and at the edge of the embedded clause, the *wh*-filler in gap position merely refreshes these elements. However, with a mismatching matrix-clause antecedent for the pronoun, e.g., W*hich decision regarding her did Paul say that Lydia rejected?,* identification with the value of the matrix subject is not possible, so the variable discourse referent for the feminine pronoun must be actively maintained as unresolved at this point in the sentence, inducing greater working memory load. In a Comp structure like W*hich decision about her did Paul say that Lydia rejected?*, the processing of the edge of the embedded clause predictively enables a nominal (*n*) placeholder syntactic binder {*n*: Gender: Feminine, *her*}, eventually identified as the embedded-clause subject *Lydia*. These differences should therefore be reflected in γ ERPDs as the tensed clause is established by the subordinator.

Dekydtspotter et al.’s (2023) study of induced oscillatory responses reported modulation in ERPDs in induced power in the low β band (13–14 Hz) early into the bridge verb presentation (0–250 ms) in L1 and L2 speakers, presumably as the verb phrase was predicted. These ERPD patterns revealed greater power for Comps than Mods in match conditions (1a, b), consistent with a need for greater resources here to compute syntactic binding than to refresh already-established discourse coreference. Additionally, the ERPDs revealed greater power for Mods than for Comps in mismatch (1c, d), as an unresolved and unbound pronoun requires active maintenance of an unidentified discourse referent, whereas an anticipated bound-variable interpretation in Comps removes that need (Koornneef and Reuland, 2016). These ERPDs were then repeated at 15–16 Hz early into the subordinator (30–367 ms) as a tensed clause was predicted. Differences between L1 and L2 speakers were also observed, with L2 speakers showing additional β power in support of anaphora resolution through binding. These ERPDs are consistent with the role of the β band in maintaining the current cognitive set and enabling prediction (Lewis et al., 2023) and with extra processing costs for L2. Early timing of ERPDs in β, before the verb could even be retrieved, challenges the notion that L2 speakers’ processing is not predictive (Covey et al., 2024). However, results in β oscillations do not as such address the processes of delayed computations of lexico-grammatical objects and anaphoric syntactic relations in L2 as per Clahsen and Felser (2018) and do not address claims of interference in retrieval from working memory (Cunnings, 2017a,b).

In sum, the patterns of γ oscillations in anaphora resolution in Nieuwland and Martin (2017) might be linked to a γ syn-sem operational workspace implementing the computation of referential chains in anaphora resolution (Kazanina and Tavano, 2023c; Murphy, 2024) in addition to sequential phrasal arrays computed in slow rhythms (Ding, 2022; Meyer et al., 2019).

### Research questions and predictions

On our hypothesis, the reinstatement of elements to the γ syn-sem operational workspace takes place in low γ, induced by an embedded tensed clause binding domain. The retrieved expressions normalized to basic elements would enable referential chain formation in broadband γ and in coactivation of cortical sites synchronized for lower and higher representational levels. Structure-dependent anaphora-linked γ ERPDs would arise in the computation of referential chain objects, as a tensed embedded clause provides a binding domain for silently re-represented pronouns in Comps (1b, d) vs. Mods (1a, c). Hence, γ ERPDs will reflect greater power for Comps (1b) vs. Mods (1a) in matrix-clause antecedent match, reflecting the implementation of anaphoric relations, with greater power for Mods (1c) vs. Comps (1d) in matrix-clause antecedent mismatch, as a referential chain object eliminates the need to maintain an unresolved discourse referent for the pronoun, reducing memory load (Koornneef and Reuland, 2016).

Therefore, turning first to the role of narrowband- vs. broadband-γ oscillations in the γ syn-sem operational workspace, RQ1 asks: Will broadband-γ ERPDs that reflect processes involved in anaphora resolution arise irrespective of L1 or L2 status? The line of thinking laid out so far suggests that they will (Claim 1). These anaphoric computations in real time determined by structural constraints are unexpected under shallow structuring in L2. RQ2 asks: Will narrowband ERPDs in low γ that reflect the reinstatement of referential elements from item memory arise irrespective of L1 or L2 status? We hypothesize that they will (Claim 2). Such across-the-board effects are unexpected under retrieval difficulties from working memory or under failure to engage in prediction. Table 1 summarizes the expectations with respect to power differences, timing, and frequencies.

**Table 1:** Expectations in power differences, timing, and frequencies.

ERPDs	Match: [(1a)-(1b)] > negative (more power for Comps) Mismatch: [(1c)-(1d)] > positive (more power for Mods)
Timing	As a binding domain arises in the construction of an embedded tense clause
Frequencies	low γ (30–50 Hz); broadband γ (30–120 Hz)

## Materials and methods

This research was approved by the Indiana University Institutional Review Board. At the start of the experimental session, participants read the study’s Statement of Informed Consent. They were asked whether they had any questions and whether they consented to participate in the study. They provided verbal consent to the researcher, in line with the approved IRB Protocol, and were reminded that they could withdraw at any point. The stimuli consisted of 200 trials. The 25 experimental quadruples representing 100 trials are exemplified in (1a-d): (1a, c) involve Mod structures; (1b, d) involve Comp structures; (1a, b) involve a matrix subject that matches the gender of the pronoun inside the *wh*-filler; and (1c, d) involve a matrix subject that does not; 50% of trials involved masculine referents/pronouns, and 50% of trials involved feminine referents/pronouns. One hundred distractor items involved complex interrogative structures and permutations like the target items, which were counterbalanced so that no grouping stood out. The stimuli appeared in four blocks presented in random order and with randomization within each block, and crucially, no two items from a set ever appeared in the same block. The greater variability inherent among L2 speakers, e.g., in vocabulary size and experience with the language, needs to be mitigated in L2 research. Hence, presenting all items in a set like (1a-d) to participants enables a representative understanding of L2 grammatical processing ability, which might otherwise be diluted by differences in lexical access difficulty across conditions. E-Prime (2016) delivered the stimuli. Each sentence appeared word by word at the center of the screen in 36-point Consolas font, using normal orthographic conventions. Participants sat in a chair facing a computer monitor approximately 4 feet away. A fixation cross at the center of the screen preceded each item, lasting 700 ms. During the stimulus presentation, each word appeared for 300 ms and was followed by a 250-ms blank screen. Due to the time required for E-prime to load each word and for the monitor’s refresh rate, the total presentation time was 566 ms (300-ms word presentation, 250-ms interstimulus interval, and a 16-ms refresh rate between words). It accommodated L2 speakers without being unnaturally slow for L1 speakers. Indeed, the task was found to be strenuous but manageable to advanced L2 speakers in stimuli preparation.

Respondents were trained to read questions like the stimuli and then complete true–false comprehension checks, which were presented in their entirety for a maximum of 3,500 ms. These comprehension checks were of several types: Some examined a pronoun’s anaphoric interpretation, while others queried other aspects of the sentences. Participants were asked to quickly respond to the statements by pressing the left arrow key for “True” and the right arrow key for “False.” There was a training session of six items, which could be repeated before moving on to the experiment. All training items were followed by a comprehension check; in the task, only two thirds were. This ratio maintained participant attention without being overly taxing. Naturally, a set of questions like our stimuli seems plausible in only a limited set of situations. Thus, respondents were introduced to a context involving two friends who were devoted followers of a television series. One of the friends, however, had missed some episodes and asked the other some questions to catch up.

### Participants and testing procedures

Following Lewis et al.’s (2016a) examination of oscillations in 20 L1 speakers and 20 L2 speakers, we selected a sample size of 48 participants, with two groups of 24. We report results from 24 L1 speakers of French (20 right-handed; 4 left-handed) and 24 L2 speakers of French (23 right-handed; 1 left-handed). Grey et al. (2017) argued that neurocognitive models of language “largely formulated around data from only right-handers” (p. 27) problematically ignore variability in neurologically healthy populations. A cortical hierarchical syn-sem operational workspace for language should hold across neuroanatomical diversity associated with handedness. Lateralization does not fully mirror the direction of handedness, as the majority of left-handers are left-lateralized for language, but the proportion of people with bilateral or right lateralization is higher in left-handers than right-handers (e.g., Woodhead et al., 2021). Furthermore, recent neurological models have argued for bilateralization in the lexical interface and combinatorial syntax-semantics mappings in speech processing (e.g., Hickok and Poeppel, 2007). However, given the long-standing practice of excluding left-handers from processing studies, the possible influence of handedness was addressed by comparing effects for the general population with effects for right-handers only.

After providing biographical information, participants completed a C-test to gauge their overall proficiency in French. A C-test involves paragraph-length texts in which the second half of every other word is removed. The C-test (Renaud, 2010) consisted of two unrelated texts with 50 partially missing words (25 content words and 25 function words) across the two paragraphs. Respondents were given 5 min per paragraph to fill in the missing parts of the words. C-tests were scored for accuracy out of 50 points. Finally, participants completed the EEG task, with each of the four blocks lasting 13 min; including breaks, the total task time was approximately 1 h. These procedures ensured that the subjects would not be fatigued and could be expected to stay engaged.

The 24 L1 speakers of French (average age = 26.6, *SD* = 4.32) were tested in the United States. They had, on average, lived abroad for 2.4 years (*SD* = 2.61) at the time of testing. The average C-test score was 48.7/50, with a range from 45 to 50. The 24 L2 speakers of French (average age = 28.8, *SD* = 6.37) began acquiring French during secondary schooling or later. These participants were graduate students and advanced undergraduate students in the United States at the time of testing. They had an average total length of stay of 1.2 years (*SD* = 0.69) in a Francophone country. C-test scores (average 45.5/50; range 33–50) clearly indicated that they were well above intermediate-level proficiency (typical score range 25–30). All participants were college-educated individuals with no history of dyslexia. Accuracy rates on factual comprehension checks show the task to be challenging: 61% for L1 speakers and 63% for L2 speakers. On comprehension checks related to anaphoric interpretation, L1 and L2 speakers alike interpreted the pronoun as referring to the gender-matched noun phrase 70% of the time. However, comprehension check accuracy of participants was not used as a filter for analysis: γ oscillations in basic referential chain formation constitute essential processes in unconscious, implicit, ongoing processing of the input. Such procedural knowledge is quite distinct from conscious judgments made by speakers regarding intended meanings or linked to longer-term memory for the entire sentence. In terms of reference queries, anaphoric interpretations, although preferred, are not solely required. Even when an anaphoric dependency is preferred, deixis is never excluded. Generally, real-time basic brain processing as participants compute a bi-clausal filler-gap dependency is expected to be independent of their behavior on comprehension checks following individual sentences.

### EEG procedures

**Figure 3:**
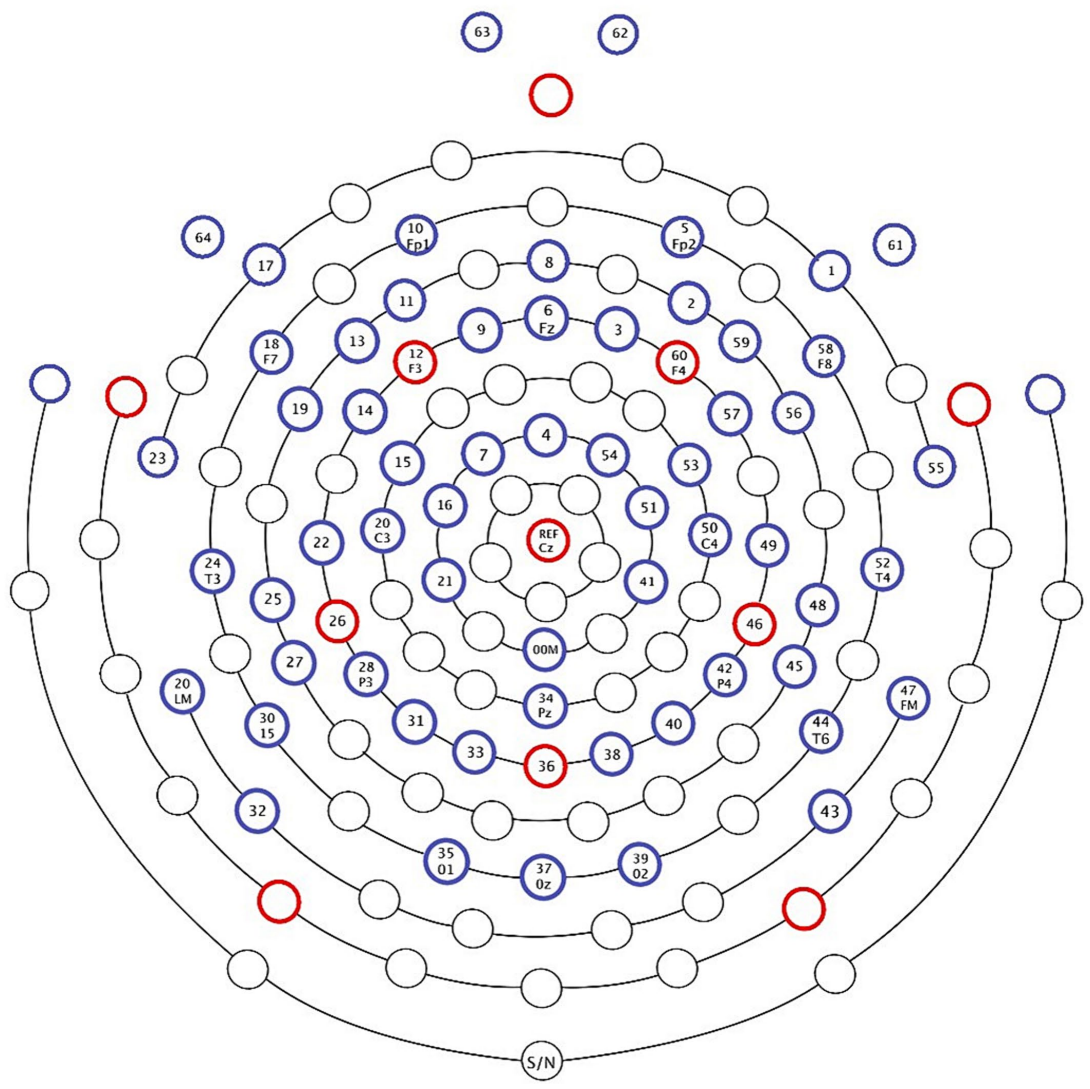
EGI 64-electrode system.

EEG was recorded at a 1,000 Hz sampling rate via a 64-electrode EGI system (Electrical Geodesics Inc., Eugene, OR; Figure 3) referenced to Cz (vertex) online. The signal was collected using a Net Amps 300 amplifier with a gain of 5,000 and acquisition software NetStation (version 4.5.4). Impedances were verified to be below 50 kΩ before each of the four blocks in the task. All preprocessing and data cleaning procedures were performed using the EEGLAB toolbox based on MATLAB (version 9.5) (Delorme and Makeig, 2004). An 8-ms latency shift due to the amplifier was corrected before preprocessing. Line noise was removed using the CleanLine plugin for EEGLAB (Mullen, 2012). The continuous data were then divided into 5.2-s epochs starting with *est-ce que* (the question marker) and running to the end of the sentence. Following segmentation, we visually inspected each epoch for bad channels, and if a channel was bad in more than 10% of epochs, we removed the whole channel. It has been demonstrated that muscle activity can create noise in high-frequency EEG measures, and γ-band results should thus be reported and interpreted with caution (e.g., Yuval-Greenberg et al., 2008). Hipp and Siegel (2013) showed that removing such artifacts from the EEG recording by rejecting data sections affected by artifactual signals or independent component analysis (ICA) can allow for a more confident analysis of high-frequency EEG. Therefore, we visually inspected each epoch and systematically removed any epoch including unexpected EMG activity (i.e., furrowing of the brow, face, and neck movements, but not blinks). We used the binica algorithm for ICA to extract and then manually check the 32 most impactful components generated by principal component analysis (PCA) so that we could effectively remove the remaining ocular and cardiac activity, among any remaining artifacts; 12 additional participants with greater than 10% bad channels or greater than 30% bad epochs were excluded from the analysis, leaving the 24 L1 and 24 L2 speakers described above. An average of 85% of trials was retained across subjects (*SD* = 2.20). The average number of trials retained was similar across conditions, (1a), *M* = 21.31, *SD* = 2.23; (1b), *M* = 21.06, *SD* = 2.02; (1c), *M* = 21.17, *SD* = 2.42; (1d), *M* = 21.27, *SD* = 2.12; *p* = 0.91, with no differences across groups (NS: *M* = 21.21, *SD* = 2.13; L2 speakers: *M* = 21.20, *SD* = 2.28, *p =* 0.93). For L1 speakers, 290 components were rejected, for an average of 12 per subject; for L2 speakers, 345 components were rejected, for an average of 14 per subject; and for the whole population, 635 components were rejected, for an average of 13 per subject. These may seem at first like high numbers of components to remove. However, our 5.2-s epochs are much longer than in most research, and longer epochs are likely to contain more noise than shorter ones. Because component removal for artifacts may risk removing some brain activity, the procedure is conservative in terms of finding effects. No significant difference arose between groups in the number of components removed (*p* = 0.35). The data were average referenced, and missing channels were interpolated for the time-frequency analysis.

### Time-frequency analysis

The preprocessed EEG data were loaded into the FieldTrip toolbox (Oostenveld et al., 2011) as eight datasets for the structural conditions (1a-1d) and two groups (L1 vs. L2). The time window of theoretical interest is constituted by the two critical bridge words *dit que* “said that,” lasting 1,132 ms, 566 ms for each word. The 750 ms prior to the onset of the target word *dit* “said” was included as a baseline period, for a total selected time window of 1,882 ms. Building on Dekydtspotter et al. (2023), which reported ERPDs in induced power in the β band, we examined γ-band activity in evoked power that reflects both time-locked and phase-locked oscillatory responses. We first calculated the ERPs of each condition for each subject. Then, we convolved a family of Morlet wavelets of seven cycles in 0.5 Hz steps with in the selected time window of each EEG trial, which yielded the time-frequency information of the neural activity. The length of the wavelets was set as three standard deviations of the Gaussian kernel. At 60 Hz, the wavelet duration was 0.037 s. The spectral bandwidth was 17.143 Hz. We log-transformed (10*log10) the derived power in FieldTrip to standardize the unit as decibels at each of the frequencies between 30 Hz and 120 Hz for each condition of each subject in each group. The transformed power data for L1 speakers and L2 speakers were used as the basis of the following statistical analysis. The data can be accessed at https://datacore.iu.edu/concern/data_sets/7w62f949g?locale=en.

### Data analysis

Our experiment followed a mixed between–within-subjects 2 × 2 factorial design for two groups. The first factor was structural conditions (Mod–Comp power differences in a matrix-clause antecedent match vs. mismatch) and the second factor was group (L1 vs. L2), with the between-subject factor being the L1 vs. L2 status. We, therefore, used a 2 × 2 factorial model simultaneously accounting for between-subjects variance and within-subjects variance for the two units of observation in the data, which allows for the detection of patterns across L1 status and L2 status as well as differences between groups. Procedures for a 2 × 2 factorial model avoid making non-statistically supported claims for superficial L1 vs. L2 differences by taking both within- and between-subjects variances into consideration simultaneously. Indeed, as Cheng et al. (2021) argued, “researchers’ constructed categories…ignore the ways in which these artificially ‘different’ groups could be similar in their perception and production of language” (p. 8).

Data were analyzed with cluster-based non-parametric permutation tests to avoid the multiple comparison problem for our medium-density electrodes, on the assumption that the spatially adjacent channels exhibit similar spectral-temporal features (Groppe et al., 2011; Larson-Hall, 2016; Maris and Oostenveld, 2007). We conducted two types of non-parametric statistical tests (paired-sample and independent-sample *t-*tests) using Monte Carlo simulations with 1,000 random samplings for each channel–frequency–time triplet. As we are interested in the broadband hierarchical processing across the γ band and the narrowband processes of retrieval in low γ frequencies, we used two bins: first, 30–120 Hz, to identify hierarchical processing in broadband γ and then 30–50 Hz (low γ), given the specific role of narrowband activity in cortical–subcortical transfer.

We calculated Mod–Comp power differences between the antecedent-match [(1a)-(1b)] and antecedent-mismatch [(1c)-(1d)] conditions to address whether distinct allocations of resources to Comps vs. Mods can be found across L1 status and L2 status. Power differences were analyzed with paired-sample *t*-tests using the maximum of the cluster *t*-test statistics with 1,000 permutations. Bonferroni correction was performed to correct the multiple comparison problem due to the two frequency bins, so the corrected alpha level is α = 0.050/2 = 0.025. We first examined γ oscillations in L1 and L2 speakers, including both left- and right-handed respondents (Grey et al., 2017). To guard against a possible effect of different lateralization patterns in the entire population, we conducted *post-hoc* analyses of the exact patterns of frequency and electrode distribution on just the right-handed respondents. This ensured that the exact patterns detected for the entire population also arose in subjects with the same handedness. Because a handedness effect might still be possible, we then re-ran the original analysis described above on just the right-handed respondents to compare with the original results. At each step, we adopted a Bonferroni protection for multiple comparisons.

To address possible L1-L2 differences, we compared Mod–Comp power differences [(1a + c)-(1b + d)] between L1 and L2 speakers using independent-sample *t*-tests using the maximum of the cluster *t*-test statistics with 1,000 permutations. We again adopted a Bonferroni correction of α = 0.025 for two bins. We also examined Conditions*Group interactions between L1 vs. L2 speaker status and Mod vs. Comp structure in match vs. mismatch. FieldTrip does not provide automated interaction estimation. Therefore, following the recommended procedures,^1^ we calculated Mod–Comp power differences between the antecedent-match [(1a)-(1b)] and antecedent-mismatch [(1c)-(1d)] conditions for each group. The dependent variable is therefore the difference between these power differences ([(1a)-(1b)]-[(1c)-(1d)]) for each group during the processing of *dit que* “said that”. Independent-sample *t*-tests based on permutations were performed on this difference of differences with a Bonferroni correction of α = 0.025 for two bins.

Across our analysis, permutation tests provide the time window and electrodes in which a significant effect arises. They, however, lack precision as to the exact timing and location of effects (Sassenhagen and Draschkow, 2019). Our discussion of the timing of ERPD effects is therefore limited to the window in which the effects are found.

## Results

**Figure 4:**
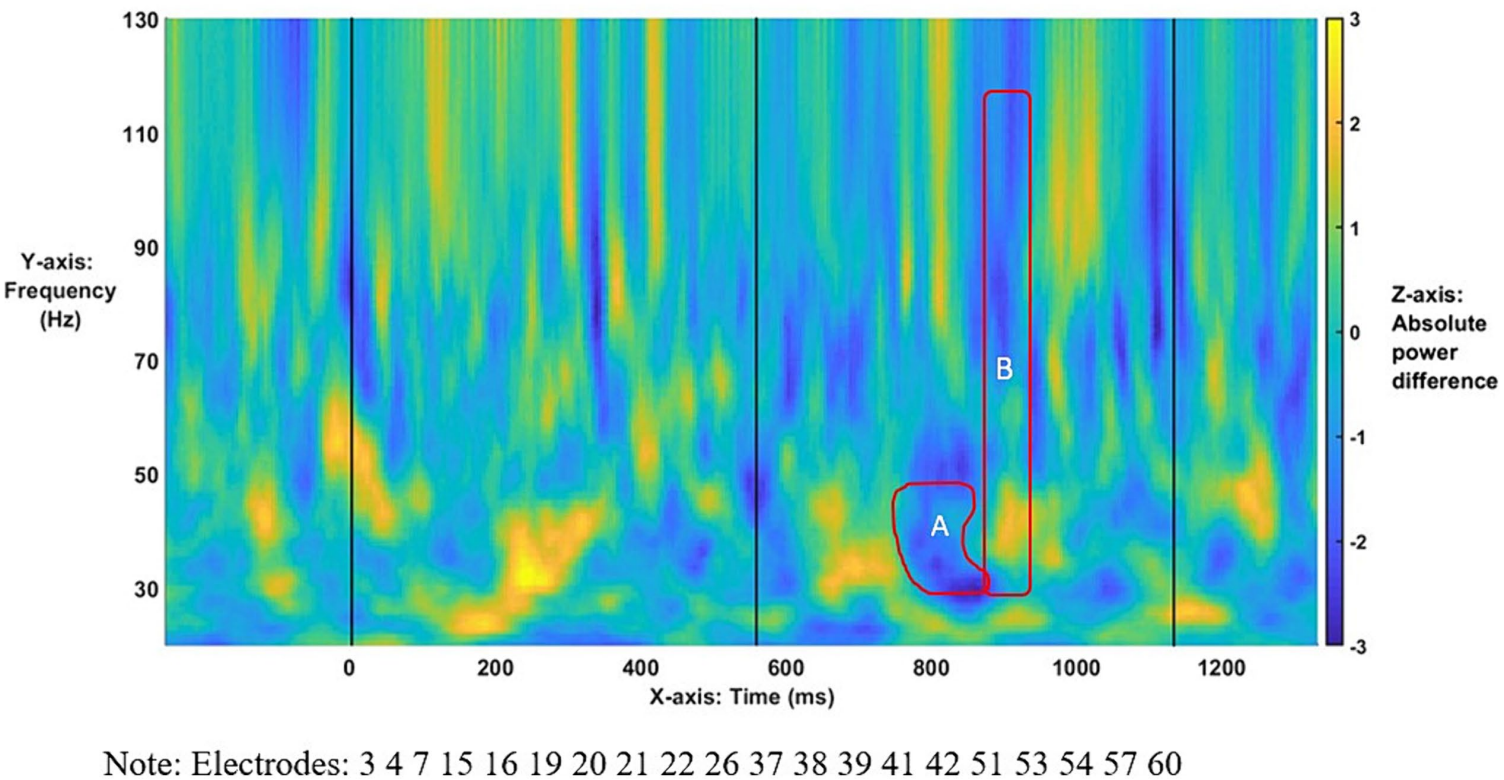
Time–frequency plot of low-γ **(A)** and broadband-γ **(B)** effects. The onset of the bridge verb occurred at “0”. The vertical lines indicate the offset of the verb and subordinator, respectively.

We first turn to the results investigating possible main effects of group and Conditions*Group interactions for L1-L2 differences. No possible main effects of group reached statistically significant levels within these bins: at 30–120 Hz, the lowest value for any cluster was *p* = 0.17, *t* = −150.76, and at 30–50 Hz, the lowest value for any cluster was *p* = 0.51, *t* = −97.64. Similarly, no statistically significant interaction of conditions and group was found either at 30–120 Hz, where the lowest value for any cluster was *p* = 0.95, *t* = −14.96, or at 30–50 Hz, with *p =* 0.72, *t* = −56.86 as the lowest value for any cluster. Anaphora-linked ERPDs were predicted to arise in evoked γ power for the entire population as a *wh*-filler was merged and integrated within an embedded-clause structure. Table 2 shows that statistically significant ERPDs were found first in low (30–50 Hz) γ (Effect A, Figure 4) and then in broadband (30–120 Hz) γ (Effect B, Figure 4) precisely as the subordinator *que* “that” introducing an embedded clause was processed. In particular, the ERPDs matched the predicted power difference patterns. That is, the difference between Mods and Comps in the antecedent-match conditions, i.e., [(1a)-(1b)], resulted in a negative value. In contrast, the difference between Mods and Comps in the antecedent-mismatch conditions, i.e., [(1c)-(1d)], yielded a positive value.

**Table 2:** ERPD effects at subordinator *que* “that” (*n* = 48).

Figure 4	Hz	*p*-value	*t*	Timing ms (duration)	Electrodes	Power differences (Mod–Comp)
Effect A	30–50	0.019	−474.33	773–853 (80)	15 16 19 20 21 22 26 37 38 39 41 42 51 53 57 60	Match: [(1a)-(1b)] = −1.4950 Mismatch: [(1c)-(1d)] = 0.8520
Effect B	30–120	0.015	−383.95	848–918 (70)	3 4 7 21 50 51 54 60	Match: [(1a)-(1b)] = −0.9169 Mismatch: [(1c)-(1d)] = 1.4945

For purely descriptive purposes, we note that these ERPD patterns were echoed at the group level, with both L1 and L2 speaker groups exhibiting greater power for Comps vs. Mods in matrix-clause antecedent-match conditions in low γ, L1, [(1a)-(1b)] = −1.0016, L2, [(1a)-(1b)] = −0.8632; and broadband γ, L1, [(1a)-(1b)] = −0.8457, L2, [(1a)-(1b)] = −1.4224; and greater power for Mods in matrix-clause antecedent-mismatch conditions in low γ, L1, [(1c)-(1d)] = 1.1005, L2, [(1c)-(1d)] = 0.8349; and broadband γ, L1, [(1c)-(1d)] = 1.5649, L2, [(1c)-(1d)] = 2.0142.

The time–frequency plot of absolute power differences for the entire population (Figure 4) shows virtually no overlap between Effects A and B (only 5 ms as shown in Table 2), indicating a narrowband effect in low γ that ends as the broadband effect across γ starts. For the broadband effect, Figure 4 suggests that the power-based differences arise largely in high-/mid-γ frequencies, as indicated by the darker blue, with lesser differences in the lower γ frequencies.

**Figure 5:**
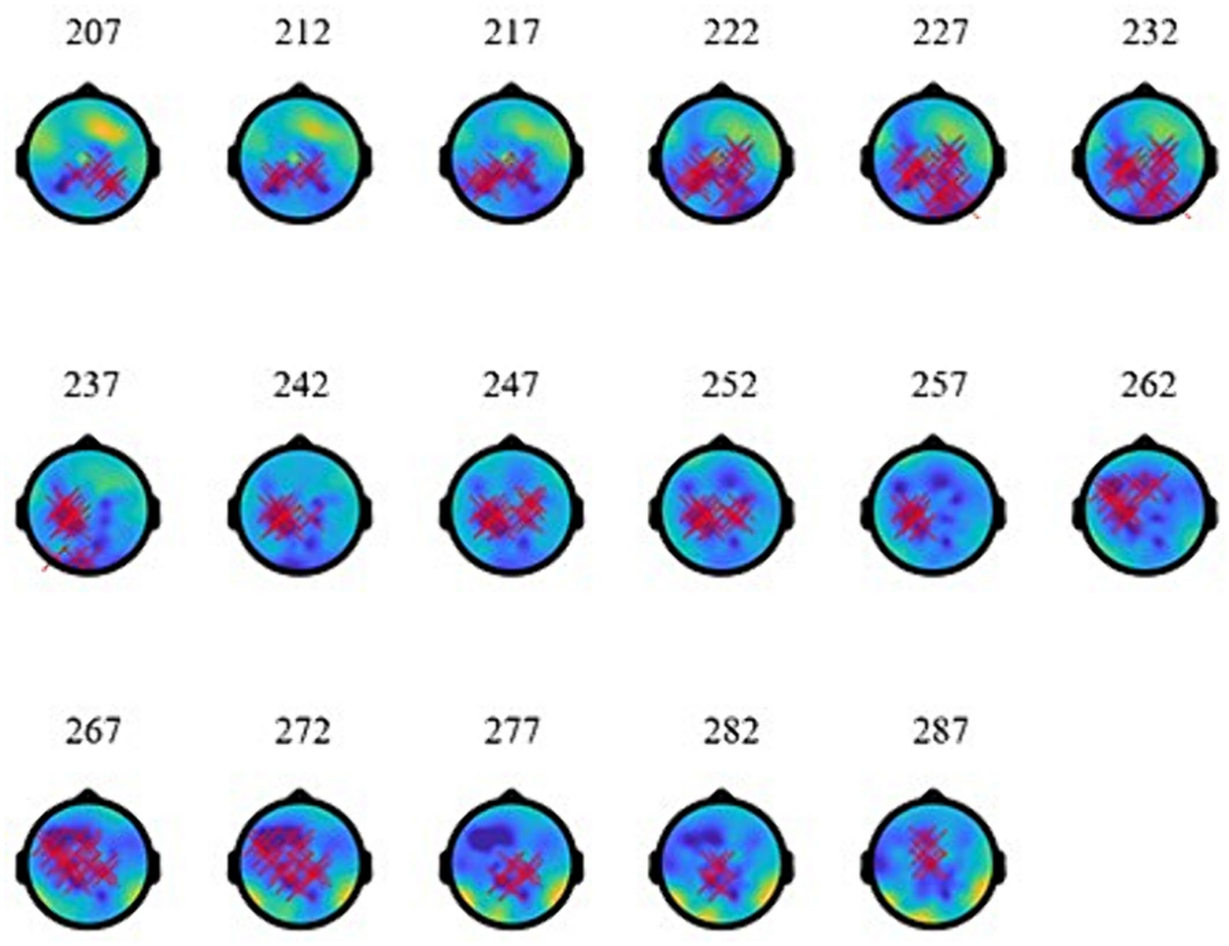
Topographies of low-γ effect **(A)** at 773–853 ms (during the subordinator). The red marks indicate electrodes where significant ERPDs were detected.

**Figure 6:**
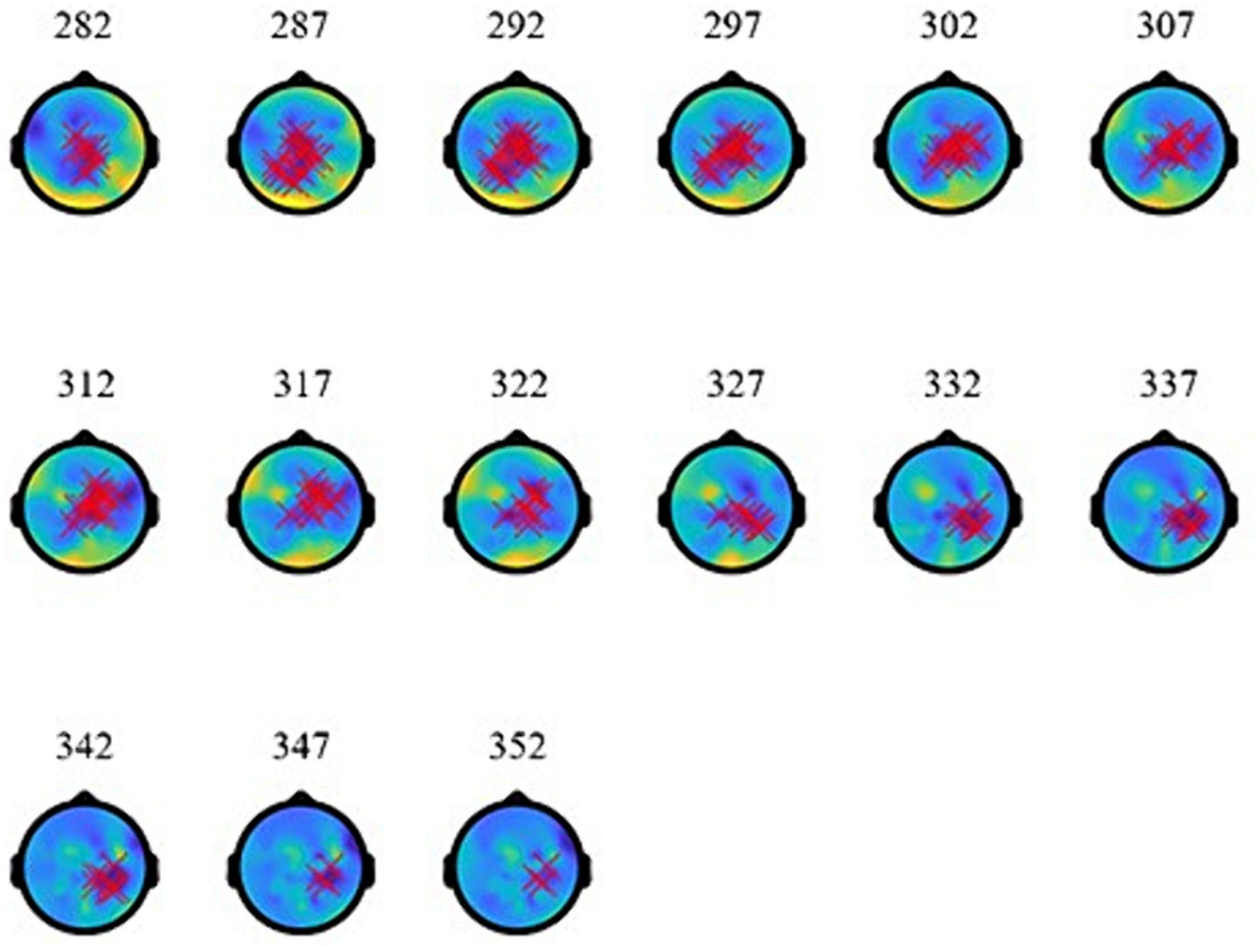
Topographies of broadband-γ effect **(B)** at 848–918 ms (during the subordinator). The red marks indicate electrodes where significant ERPDs were detected.

As the topographies in Figure 5 show, the narrowband Effect A in low γ arose across bi-hemispheric centro-posterior electrodes and reached temporal and anterior electrodes (marked in red). The topographies in Figure 6 show that the broadband Effect B across γ has a bi-hemispheric centro-posterior distribution that moves to parietal right-hemisphere electrodes over time. The distinct distributions in frequency, time, and space for the ERPD effects are compatible with each effect signaling a different function.

**Figure 7:**
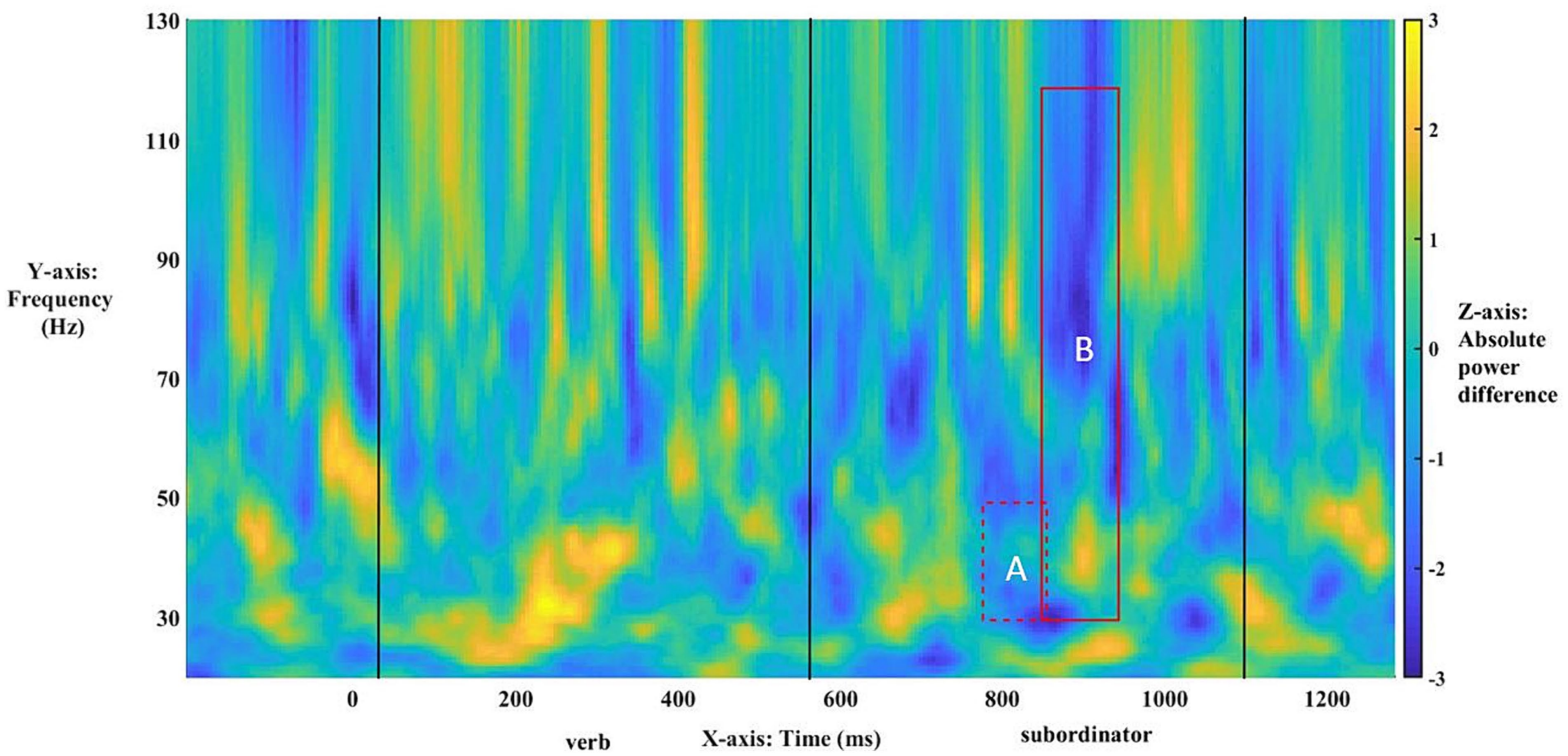
Time–frequency plot of low-γ **(A)** and broadband-γ **(B)** effects among right-handers only. The onset of the bridge verb occurred at “0”. The vertical lines indicate the offset of the verb and subordinator, respectively.

*Post-hoc* analyses of these exact patterns of frequency and electrode distribution obtained for the entire population were conducted with just the right-handed respondents. They showed the same statistically significant effects across low γ (30–50 Hz), *t*(42) = −3.7493, *p* < 0.001, and broadband γ (30–120 Hz), *t*(42) = −3.9658, *p <* 0.001, Figure 7. A re-running of the original analysis only on right-handed participants strongly echoed the broadband effect for the entire population in terms of timing and asymmetries: The exclusion of left-handed individuals boosted broadband effects across γ (Table 3, Figure 7). The narrowband effect in low γ, while confirmed in the *post-hoc* test above, was not significant in this procedure, likely due to loss of power. Across the statistically significant effects for the entire population and right-handed respondents only, the power asymmetries responsible for the ERPDs were similar: greater power for selected Comps vs. unselected Mods in matrix-clause antecedent-match conditions and greater power for Mods in matrix-clause antecedent-mismatch conditions.

**Table 3:** ERPD effects at subordinator *que* “that” in right-handed participants (*n* = 43).

Figure 7	Hz	*p*-value	*t*	Timing ms (duration)	Electrodes	Power differences (Mod–Comp)
Effect A	30–50	0.3	−142.54	773–853 (80)	15 16 19 20 21 22 26 37 38 39 41 42 51 53 57 60	Match: [(1a)-(1b)] = −0.4520 Mismatch: [(1c)-(1d)] = 0.8904
Effect B	30–120	0.006*	−465.97	848–923 (75)	2 16 21 50 51 56 59 60	Match: [(1a)-(1b)] = −0.8050 Mismatch: [(1c)-(1d)] = 1.3633

*Indicates a statistically significant result following Bonferroni correction (α = 0.025).

The specific anaphora-linked γ ERPDs across the entire population reported here echoed the ERPDs in Dekydtspotter et al. (2023) in the low β band, which were consistent with the view that β actively tracks sentence-level representations (Lewis et al., 2023), thereby enabling temporal predictions. However, γ-band activity more likely supports basic syn-sem operations in language computations (Kazanina and Tavano, 2023c; Murphy, 2024). Moreover, the asymmetries are consistent with the idea that Comps enable a syntactically guided bound-variable interpretation of the pronoun. This enables anaphora resolution in the antecedent-match condition and removes the need to maintain an unidentified pronominal referent in discourse in the antecedent-mismatch condition.

## Discussion

Mod–Comp ERPDs in a match vs. mismatch arose in evoked γ power as the subordinator *que* “that” was processed across L1 and L2. RQ1 asked whether anaphora-linked ERPDs would arise in broadband γ across L1 and L2. RQ2 asked whether narrowband ERPDs in low γ that reflect the reinstatement of referential elements from item memory would arise irrespective of L1 or L2 status. Both research questions can be answered affirmatively. These narrowband and broadband ERPDs matched expectations with respect to power differences, timing, and frequencies as per Table 1, reflecting distinct steps in the implementation of anaphoric relations for Mods and Comps in antecedent-match and antecedent-mismatch conditions. ERPDs first arose in low-γ power, presumably as cell assemblies formed when referential elements were returned from working memory storage to the hierarchical γ syn-sem operational space to participate in referential chain formation in anaphoric binding (Figure 2). The timing of these ERPD effects, therefore, matched the hypothesis that the subordinator’s requirement for a tensed clause—i.e., a subject and verb phrase defining a binding domain per Binding Condition B (Chomsky, 1981)—constrains referential relations across L1 and L2. The power-difference values for each group in the two bins echoed the power-difference values accounting for the main effect of structure across groups in each bin. We proposed that broadband-γ effects arise as sites for higher and lower representation levels synchronized in γ are coactivated within temporal circuitry implementing a hierarchical syn-sem operational workspace, wherein referential elements are combined and a discourse referent is assigned, implementing syntactic binding.

Our findings about anaphoric relations dependent on silent *wh*-fillers in long-distance dependencies support Kazanina and Tavano’s (2023a) “idea of implementing recursion through a two-level abstract chunking structure and a backward loop from the lower to the higher level” (p. 724). Furthermore, we argued that a processing loop as modeled following Murphy’s (2024) ROSE architecture (Figure 2) can help address Coopmans et al.’s (2023) concern about “how ‘relational’ information, as found in long-distance dependencies, could be extracted” (p. 723). The observed processing differences between Mod and Comp structures with matrix subjects that either matched or mismatched the pronouns inside the *wh*-filler speak to the interaction between working memory and parsing. Greater working memory is required when a variable discourse referent cannot be immediately identified. The role of working memory does not impede the argument that basic structure-dependent hierarchical syn-sem operations in referential chain formation are implemented in γ. Indeed, referential relations linked to silent re-representations of previously stored *wh*-expressions during the processing of clause-edge sequential arrays point to specific abstract hierarchical computations that differ based on the fundamental Mod vs. Comp difference. The anaphora-linked γ ERPD patterns in Table 2 arose as a binding domain that was enabled in *wh*-filler re-representation in the absence of concomitant differences in the sentential input, indicating computations of coreference vs. structure-based binding in γ oscillations. This points to a γ syn-sem operational workspace implementing basic referential chain objects in the ongoing processing of the sentence as in Figure 2.

With respect to implicit knowledge of L1 vs. L2, the acquisition of French by L1-English speakers must include the establishment of new local brain microcircuits for grammatical gender. Local circuits for new features are distinguished from feature-specific local circuits already instantiated in the L1, as suggested by behavior in speeded judgment tasks (Lopéz Prego and Gabriele, 2014) and delayed P600 ERP effects (Gabriele et al., 2013; Lemhöfer et al., 2014). In the current study, ERPD effects across the L1 vs. L2 distinction suggest that whether feature-specific local circuits responding to the input were generated in L1 or L2 acquisition, the hierarchical syn-sem computational mechanism in γ seems to be fundamentally the same. Not finding statistically significant differences between groups within these bins does not exclude the possibility of differences for feature-specific local circuits acquired in L1 or L2 acquisition. Indeed, L1 vs. L2 effects in β reported in Dekydtspotter et al. (2023) show downstream differences in at least the processes subserving the maintenance of the ongoing sentence interpretation. Similarly, Lewis et al. (2016a) reported β-range effects for L2 speakers in gender processing that are suggestive of the role of this rhythm in processing load management. However, the mechanisms implementing basic syn-sem operations in retrieval and in the construction of referential chain objects for hierarchical syntactic structures with abstract elements do not appear to be significantly impaired or majorly delayed (*cf.* Clahsen and Felser, 2018; Cunnings, 2017a,b). A dynamic processing loop also suggests the built-in anticipatory nature of language processing in L1 and L2 speakers (*cf*. Covey et al., 2024). The evidence provided by γ oscillations therefore calls into question a series of hypotheses about L1 vs. L2 processing, while supporting the idea of a similar mechanistic process with different resource allocations in the maintenance of the processing.

### Epistemology, language operations, and hierarchical γ-band processing

Chomsky (2020) noted: “each language constructs in the mind an infinite array of structured expressions each of which has a semantic interpretation that expresses a thought, each of which can be externalized in one or another motor system” (p. 17). As Berwick and Chomsky (2016) pointed out, structural hierarchy as the root of knowledge of the language is discernible in interpretive ambiguities like *Flying planes can be dangerous*—where either the activity of flying planes can be dangerous or planes in the air can be dangerous. This ambiguity for the same sequential array *flying planes* requires alternative structures with verbal and nominal heads and related semantics. In an operations and structures loop architecture, a hierarchical γ cortical workspace implementing basic operations across syntactic and semantic features represented in the cortex enables phrasal structures in subcortical slow rhythms (Kazanina and Tavano, 2023c; Murphy, 2024). Overall, hierarchical computations in broadband γ with narrowband processes in low-γ sites recursively interacting with subcortical sites synchronized in slow rhythms avoids potential problems associated with a strict layering of rhythms in structural hierarchy (Kazanina and Tavano, 2023c). Investigating a computational workspace for Merge, Adger (2017) found that such design rules out a range of illicit derivations (such as parallel/sideways derivations) without the need to specify constraints to rule these out. Hence, aspects of language would result from a neurocognitive design including a cortical hierarchical γ syn-sem workspace that implements anaphoric chain relations, among others, in terms of more basic operations such as the combination of referential expressions of different sizes and/or structures and value assignment (Figure 2). The processing loop associated with this architecture can also account for aspects of real-time processing (Crocker, 1996; Fodor, 1998; Stabler, 1991; and many others). Similarly, the results suggest that the same general mechanistic γ-band processes interact with feature-specific local brain microcircuits to generate semantically interpreted objects and structures, irrespective of whether these feature-specific local brain microcircuits were established in response to L1 or L2 input. Hence, in a bilingual, two such partially overlapping neurofunctional systems construct arrays of interpreted structures in the same way, accounting for similar fundamental language characteristics in L1 and L2 (Paradis, 2004).

Past empirical research on γ oscillations highlighted a role for γ in syntax and semantics, including reference, without specifying “the representations and computations that are executed by the implementational circuitry” (Poeppel, 2012, p. 52), let alone addressing their information-processing requirements. Thus, Murphy (2021, 2024) explored coupling as a mechanism of neuronal connectivity in support of information transfer across (sub)rhythms. Advancing how cortical γ computations produce language elements also requires establishing the nature of information-processing steps across domains. The processing of *the* and *man* to generate the interpreted expression *the man* requires coactivations between neuronal ensembles coding the syntactic and semantic objects for *the man* and lower levels of representations for *the* and *man*. Crucially, such hierarchical cortical processing must first combine elements across syntax and semantics and then provide a classification for each type of combination. Hence, syntactic categories such as Determiner (D) for *the* and nominal (n) for *man* combine to form {D, *n*} and similarly their linked semantic elements. Thus, the functor The, which picks out the unique contextual entity described by a nominal property, and the property Man, which picks out the set of men in the situation, combine to form {The, Man}. A value for each combination is assigned according to each domain. A category value for the syntactic combination and referential value for the semantic combination enables an interpreted object that can then be externalized. Hence, a single hierarchical process across linked domains for syntax and semantics suffices to define Merge in syntax and Function Application for functions and arguments in semantics.

## Conclusion

Echoing Kazanina and Tavano (2023c), Murphy’s (2024) ROSE architecture provides a neurocognitive model for the study of oscillations enabling language. ROSE aligns with other hypotheses in neuroscience and linguistics as well as efforts to tie linguistic processes to the biology of language (Boeckx and Theofanopoulou, 2018; Murphy, 2021). Considering this architecture for syntax, we modeled how binding relations dependent on multicycle *wh*-dependencies could be computed by simple implementational cortical circuitry defining a hierarchical γ syn-sem operational workspace. We uncovered structure-dependent anaphora-linked ERPD patterns in evoked γ power as cell ensembles formed in the implementation of syntactic binding enabled by Comps versus Mods. These effects were crucially timed with the establishment of binding domains associated with the tensed embedded clause. These effects arose across L1 and L2 speakers. These effects align with a hierarchical γ syn-sem operational workspace implementing movement and referential relations between expressions of various sizes as part of a two-level processing loop (Figure 2). We certainly do not claim that such an alignment can firmly establish our modeling of anaphoric relations beyond a reasonable doubt, but we think that this evidence opens an avenue for continued explorations along the lines introduced here. To boot, such effects arising across L1 and L2 strongly suggest that the study of oscillations within a mechanistic approach to language generation can provide additional insights into the nature of both L1 and L2 in the mind/brain. These effects are supportive of the neurobiological hypothesis that “a second language will utilize existing devices” (Green and Abutalebi, 2007, p. 560) so that circuity implementing syn-sem operations interacts with local brain microcircuits for features and feature sets that have been established in L1 or L2 acquisition to account for a single-language epistemology across learning contexts.

## Data Availability

The datasets presented in this study can be found in online repositories. The names of the repository/repositories and accession number(s) can be found at: https://datacore.iu.edu/concern/data_sets/7w62f949g?locale=en.

## Footnotes

^1^ https://www.fieldtriptoolbox.org/faq/how_can_i_test_an_interaction_effect_using_cluster-based_permutation_tests/
